# Epithelial cell identity in hyperplastic precursors of breast cancer

**DOI:** 10.1186/s40880-015-0004-z

**Published:** 2015-03-05

**Authors:** Danila Coradini, Patrizia Boracchi, Saro Oriana, Elia Biganzoli, Federico Ambrogi

**Affiliations:** Department of Clinical Sciences and Community Health, Medical Statistics, Biometry and Bioinformatics, University of Milan, Via Vanzetti 5, Milan, 20133 Italy; Senology Center, Ambrosiana Clinic, Cesano Boscone, Milan, 20090 Italy

**Keywords:** Mammary epithelial cell identity, Hyperplastic enlarged lobular unit, Atypical ductal hyperplasia, Ductal carcinoma *in situ*

## Abstract

**Introduction:**

In the adult human breast, hyperplastic enlarged lobular unit (HELU) and atypical ductal hyperplasia (ADH) are two common abnormalities that frequently coexist with ductal carcinoma *in situ* (DCIS). For this reason, they have been proposed as the early steps in a biological continuum toward breast cancer.

**Methods:**

We investigated *in silico* the expression of 369 genes experimentally recognized as involved in establishing and maintaining epithelial cell identity and mammary gland remodeling, in HELUs or ADHs with respect to the corresponding patient-matched normal tissue.

**Results:**

Despite the common luminal origin, HELUs and ADHs proved to be characterized by distinct gene profiles that overlap for 5 genes only. While HELUs were associated with the overexpression of progesterone receptor (*PGR*), ADHs were characterized by the overexpression of estrogen receptor 1 (*ESR1*) coupled with the overexpression of some proliferation-associated genes.

**Conclusions:**

This unexpected finding contradicts the notion that in differentiated luminal cells the expression of estrogen receptor (ER) is dissociated from cell proliferation and suggests that the establishing of an ER-dependent signaling is able to sustain cell proliferation in an autocrine manner as an early event in tumor initiation. Although clinical evidence indicates that only a fraction of HELUs and ADHs evolve to invasive cancer, present findings warn that exposure to synthetic progestins, frequently administered as hormone-replacement therapy, and estrogens, when abnormally produced by adipose cells and persistently present in the stroma surrounding the mammary gland, may cause these hyperplastic lesions.

## Background

According to the model proposed for breast cancer progression, normal epithelial cells progressively accumulate molecular alterations that result in a series of histologically identifiable, even if non-obligate, cancer precursors. Among the precursors earliest in the progression is hyperplastic enlarged lobular unit (HELU), a common abnormality in the adult female human breast [1]. HELU consists of an abnormal enlargement (often up to 100-fold) of normal terminal duct lobular unit (TDLU) from which it evolves. Histologically, the epithelial cells that line these dilated lobules appear increased in number and changed in morphology with the transformation of low cuboidal epithelium to irregular tall columnar epithelium. The transformation may be associated with atypia, which is characterized by the presence of round to ovoid nuclei not perpendicularly oriented to the basement membrane, and an increased ratio of nuclear to cytoplasmic [2].

Observational studies have indicated that HELU frequently coexists with more complex proliferative lesions such as atypical ductal hyperplasia (ADH) and low-grade ductal carcinoma *in situ* (DCIS) [3], suggesting it occurs as the first step in the biological continuum toward breast cancer [4]. For this reason, the presence of HELU has been proposed as a potential valuable risk factor to be thoroughly investigated and monitored [5], though clinical evidence has indicated that only a small fraction of HELUs actually progress to invasive breast cancer [6].

Among the early events required for neoplastic transformation of a mammary epithelial cell, there are perturbations in *cell type identity.* This term refers to the predetermined, tissue-specific set of genes that characterizes every cell within a multicellular organism. These genes are conserved during DNA replication and mitosis so that daughter cells retain the differentiated cell type of the parental cell (*cell memory*). Preservation of cell identity depends on a class of proteins that are collectively termed maintenance proteins [7]. Studies have shown that maintenance proteins, among which Trithorax- and Polycomb-group proteins are the best characterized, act in a somatically heritable but DNA-independent manner (i.e., epigenetically) and regulate gene expression through DNA methylation, histone modification, and chromatin remodeling [8,9]. Because of their recognized role in the control of cell identity and mammary gland remodeling, perturbations in maintenance protein expression can affect cell identity and trigger neoplastic transformation [10].

Here we used publicly accessible microarray datasets to compare, in a series of HELUs from noncancerous adult breasts and corresponding patient-matched, normal TDLUs, the expression pattern of genes experimentally recognized to be involved in regulating cell identity and mammary gland remodeling. Furthermore, in agreement with the hypothesis that HELU may represent the biological precursor of ADH to be used for the early assessment of breast cancer risk, we compared the gene expression profile of HELU with that of a series of putative invasive breast cancer precursors, namely ADH and DCIS, and corresponding patient-matched, histologically normal tissue.

## Methods

### Tissue samples

As described in the original article by Lee *et al*. [11], well-developed, single-layer type HELUs with minimal nuclear atypia and paired normal TDLUs were derived from 8 women's noncancerous breast tissues. In each formalin-fixed, paraffin-embedded tissue biopsy, HELUs and TDLUs were isolated by laser capture microdissection. Gene expression was determined using U133-X3P Human GeneChip oligo-based microarray (Affymetrix, Santa Clara, CA, USA) and the corresponding microarray dataset was publicly available at the ArrayExpress web site (http://ebi.ac.uk/arrayexpress/) under accession number E-GEOD-7377.

To compare the gene expression profile of HELUs with that of ADHs and DCISs, we used another publicly accessible dataset containing microarray data from coexisting patient-matched samples of ADH and DCIS lesions and corresponding histologically normal tissue, isolated by laser capture microdissection from surgical specimens of untreated patients with estrogen receptor (ER)-positive (immunohistochemically evaluated) sporadic breast cancer [12]. Gene expression was determined using HG-U133A Human GeneChip microarray (Affymetrix) and the corresponding microarray dataset was available at the ArrayExpress web site under accession number E-GEOD-16873.

### Gene set selection

After an extensive literature review, we established a panel of 369 genes that included genes associated with establishing and maintaining cell identity and with mammary gland remodeling, or that were involved in cell fate decision, cell growth control, cell polarity and adhesion, and steroid and transforming growth factor β (TGF-β) signaling. Of these 369 genes, 8 had no corresponding probe sets on the U133-X3P GeneChip and 40 had no corresponding probe sets on the HG-U133A GeneChip. Therefore, for the E-GEOD-7377 dataset, the gene set was actually composed of 361 elements for 842 Affymetrix probe sets, whereas for the E-GEOD-16873 dataset, the gene set was actually composed of 329 elements for 639 Affymetrix probe sets. A total of 326 genes were present in both datasets. GeneAnnot system v2.2 (http://bioinfo2.weizmann.ac.il/geneannot/) provided information about the quality of connection between each probe set and the corresponding gene in terms of sensitivity and specificity [13]. Specifically, the sensitivity, defined as the fraction of probes in a probe set that match a respective gene, is the number of matching probes in a given probe set divided by the total number of probes in this probe set. The specificity indicates to what extent the probes of a probe set bind to genes, and it sums the number of probes that match a given gene (assigning lower weight to probes that match additional genes) divided by the total number of probes that match additional genes in a specific manner.

### Statistical analysis

Because some genes are recognized by more than a single probe set, each of which is characterized by an individual specificity and sensitivity that uniquely contribute to the gene expression value, a mean gene expression value was calculated after weighting each probe set for its own sensitivity and specificity, prior to the analysis. Namely, each expression value was multiplied by the semi sum of the sensitivity and specificity of the corresponding probe set. The differential gene expression between HELUs and TDLUs and among histologically normal tissue, ADHs, and DCISs was evaluated using analysis of variance after correction for multiple testing. To correct for multiple testing, the false discovery rate (FDR) with a cut-off of 0.1 was used [14]. All analyses were performed using the open source software R 2.11.1 packages High Dimensional Molecular Data (HDMD; http://www.R-project.org).

## Results

### Genes differentially expressed in HELUs

**Figure 1:**
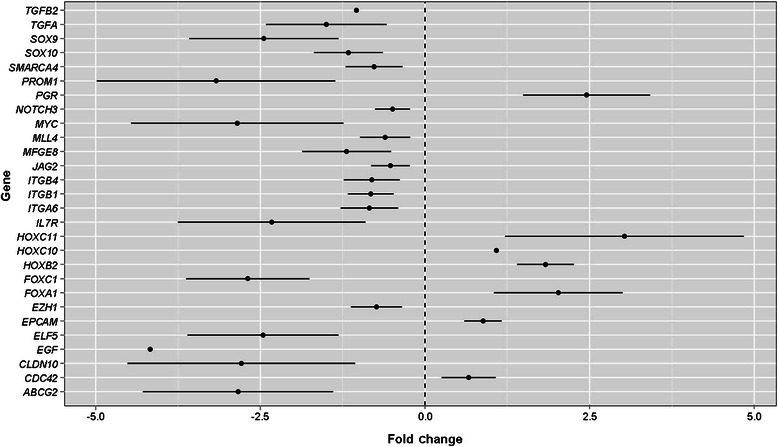
**Forest plot of the genes differentially expressed between hyperplastic enlarged lobular units (HELUs) and corresponding normal terminal duct lobular units (TDLUs) with a false discovery rate (FDR) < 0.1.** For each gene, the change is expressed as fold change ± 95% confidence interval (CI).

Compared with normal TDLUs, HELUs showed differential expression of only 28 genes (FDR < 0.1), the majority of which were underexpressed. As shown in Figure 1, the subset included genes encoding proteins typically involved in cell fate decision (*ABCG2*, *JAG2*, *MYC*, *NOTCH3*, *PROM1*, *SOX9*, and *SOX10*), embryonic development (*HOXB2*, *HOXC10*, and *HOXC11*), cell differentiation (*ELF5*, *FOXA1*, and *PGR*), epigenetic control of gene transcription (*EZH1*, *MLL4*, and *SMARCA4*), and cell adhesion (*CDC42*, *CLDN10*, *EPCAM*, *ITGA6*, *ITGB*, and *ITGB4*). In addition, the subset included some genes involved in the TGF-β signaling pathway (*FOXC1* and *TGFB2*) or encoding for growth factors (*EGF*, *MFGE8*, and *TGFA*) or a cytokine receptor (*IL7R*).

### Genes differentially expressed in ADHs and DCISs

**Figure 2:**
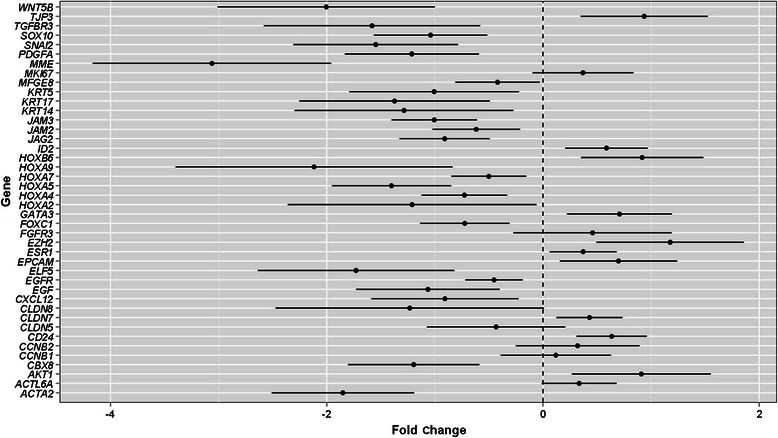
**Forest plot of the genes differentially expressed between atypical ductal hyperplasia (ADH) (upper panel) or ductal carcinoma** ***in situ*** **(DCIS) (lower panel) and histologically normal (HN) tissue with a FDR <0.1.** For each gene, the change is expressed as fold change ± 95% CI.

With respect to histologically normal tissue, ADHs and DCISs were associated, respectively, with 28 and 41 differentially express genes (FDR < 0.1), as shown in Figure 2. Notably, all genes differentially expressed in ADHs were also differentially expressed (in a similar manner) in DCISs, corroborating the hypothesis that ADH is the biological precursor of DCIS.

Most of the genes in common between ADHs and DCISs encoded proteins involved in cell fate decision (*JAG2* and *SOX10*)*,* embryonic development (*HOXA4*, *HOXA5*, *HOXA7*, *HOXA9*, and *HOXB6*), cell differentiation (*ELF5* and *GATA3*) and organization (*ACTA2*, *CD24*, *CLDN7*, *JAM2*, *JAM3*, *KRT17*, *MME*, and *TPJ3*), and epigenetic control of gene transcription (*CBX8* and *EZH2*). The set also included genes involved in the canonical and non-canonical TGF-β signaling pathways (*AKT1*, *FOXC1*, *ID2*, *SNAI2*, *TGFBR3*, and *WNT5B*) or encoding for growth factors (*EGF* and *PDGFA)* or a growth factor receptor (*EGFR)*. An additional 13 genes were specifically associated with DCISs. The set included *ACTL6A* that encodes a Trithorax protein (Baf53A) with regulatory activity in several ATP-dependent chromatin-remodeling complexes*,* 2 members of the claudin adhesion molecule family (*CLDN5* and *CLDN8*), 1 epithelial cell adhesion molecule (*EPCAM*)*,* 2 basal phenotype-associated keratins (*KRT5* and *KRT14*), 1 ER (*ESR1*), 2 cyclins involved in the G_2_/M-phase transition of cell cycle (*CCNB1* and *CCNB2)*, 1 cell proliferation-associated antigen (*MKI67*), 1 chemokine (*CXCL12*), 1 growth factor (*MFGE8*), and 1 growth factor receptor (*FGFR3*).

### Genes differentially expressed in HELUs and ADHs/DCISs

To assess the hypothesis that HELU is the putative biological precursor of ADHs and DCISs, we compared the panel of genes differentially expressed in HELUs with that of genes differentially expressed in ADHs/DCISs. As shown in Table 1, HELUs and ADHs/DCISs shared a very small subset of genes, which included *EGF*, *ELF5*, *FOXC1*, *JAG2*, and *SOX10*, all underexpressed with respect to the corresponding normal tissue.

**Table 1:** **Genes differentially expressed in HELUs versus TDLUs and ADH/DCIS versus HN tissue with a FDR < 0.1**

**Gene symbol**	**Gene name**	**HELUs vs. TDLUs**		**ADH/DCIS vs. HN**	
		**Fold change (95% CI)**	**FDR**	**Fold change (95% CI)**	**FDR**
*Common between HELUs and ADH/DCIS*					
*EGF*	Epidermal growth factor	−4.17 (ND, ND)	0.019	−1.04 (−1.71, −0.37)	0.034
*ELF5*	E-74-like factor 5	−2.46 (−3.60, −1.31)	0.058	−1.88 (−2.75, −1.00)	0.005
*FOXC1*	Forkhead box C1	−2.69 (−3.63, −1.76)	0.023	−0.95 (−1.38, −0.51)	0.004
*JAG2*	Jagged 2	−0.53 (−0.82, −0.23)	0.078	−0.92 (−1.37, −0.48)	0.006
*SOX10*	SRY (sex determining region Y)-box 10	−1.16 (−1.69, −0.64)	0.058	−1.38 (−1.92, −0.85)	0.001
*Exclusive to HELUs*					
*ABCG2*	ATP-binding cassette, sub-family G, member 2	−2.84 (−4.28, −1.39)	0.073	−0.62 (−1.20, −0.04)	0.149
*CDC42*	Cell division cycle 42	0.66 (0.25, 1.07)	0.091	0.99 (−0.12, 0.31)	0.914
*CLDN10*	Claudin 10	−2.70 (−4.52, −1.06)	0.091	0.33 (−0.26, 0.93)	0.864
*EPCAM*	Epithelial cell adhesion molecule	0.88 (0.60, 1.17)	0.019	0.14 (−0.42, 0.69)	0.928
*EZH1*	Enhancer of zeste homolog 1	−0.74 (−1.12, −0.35)	0.073	0.12 (−0.35, 0.58)	0.928
*FOXA1*	Forkhead box A1	2.03 (1.05, 3.00)	0.064	0.48 (−0.28, 1.25)	0.855
*HOXB2*	Homeobox B2	1.83 (1.40, 2.26)	0.008	−0.07 (−0.72, 0.58)	0.959
*HOXC10*	Homeobox C10	1.09 (ND, ND)	0.091	0.22 (−0.30, 0.74)	0.914
*HOXC11*	Homeobox C11	3.03 (1.22, 4.84)	0.091	0.13 (−0.15, 0.42)	0.913
*IL7R*	Interleukin 7 receptor	−2.33 (−3.75, −0.90)	0.091	0.23 (−0.38, 0.84)	0.914
*ITGA6*	Integrin, alpha 6	−0.85 (−1.28, −0.41)	0.073	−0.27 (−1.15, 0.60)	0.914
*ITGB1*	Integrin, beta 1	−0.82 (−1.17, −0.48)	0.049	0.58 (0.12, 1.04)	0.830
*ITGB4*	Integrin, beta 4	−0.81 (−1.23, −0.38)	0.073	−0.02 (−0.34, 0.30)	0.960
*MFGE8*	Milk fat globule-EGF factor 8	−1.19 (−1.87, −0.52)	0.078	−0.20 (−0.60, 0.20)	0.899
*MLL4*	Myeloid/lymphoid or mixed-lineage leukemia 4	−0.61 (−0.99, −0.22)	0.092	0.04 (−0.60, 0.68)	0.960
*MYC*	v-Myc myelocytomatosis viral oncogene homolog (avian)	−2.85 (−4.46, −1.24)	0.078	−0.45 (−1.19, 0.28)	0.855
*NOTCH3*	Notch homolog 3	−0.49 (−0.76, −0.23)	0.077	0.04 (−0.22, 0.30)	0.959
*PGR*	Progesterone receptor	2.46 (1.49, 3.42)	0.038	0.13 (−1.21, 1.46)	0.959
*PROM1*	Prominin 1	−3.17 (−4.98, −1.36)	0.078	−0.78 (−1.96, 0.40)	0.849
*SMARCA4*	SWI/SNF-related, matrix-associated, actin-dependent regulator of chromatin, subfamily a, member 4	−0.77 (−1.21, −0.34)	0.078	0.11 (−0.20, 0.42)	0.914
*SOX9*	SRY (sex determining region Y)-box 9	−2.45 (−3.58, −1.31)	0.058	−0.26 (−1.01, 0.49)	0.914
*TGFA*	Transforming growth factor, alpha	−1.50 (−2.41, −0.58)	0.091	−0.08 (−0.42, 0.25)	0.928
*TGFB2*	Transforming growth factor, beta 2	−1.04 (ND, ND)	0.092	−0.14 (−0.71, 0.42)	0.928
*Exclusive for ADH/DCIS*					
*ACTA2*	Actin, alpha 2	−0.32 (−0.71, 0.06)	0.353	−2.25 (−2.95, −1.55)	0.0001
*AKT1*	v-Akt murine thymoma viral oncogene homolog 1	0.21 (−0.65, 1.08)	0.798	1.07 (0.41, 1.72)	0.029
*CBX8*	Chromobox homolog 8	−0.82 (−1.55, −0.08)	0.207	−0.97 (−1.58, −0.37)	0.029
*CD24*	CD24 molecule	0.29 (−0.89, 1.46)	0.799	0.65 (0.34, 0.95)	0.006
*CLDN7*	Claudin 7	0.24 (ND, ND)	0.789	0.49 (0.17, 0.82)	0.036
*EGFR*	Epidermal growth factor receptor	−0.65 (−1.20, −0.09)	0.184	−0.62 (−0.90, −0.34)	0.004
*EZH2*	Enhancer of zeste homolog 2	0.90 (−0.31, 2.12)	0.412	1.61 (0.87, 2.34)	0.004
*GATA3*	GATA-binding protein 3	1.12 (ND, ND)	0.136	0.95 (0.47, 1.43)	0.008
*HOXA4*	Homeobox A4	−1.13 (−1.92, −0.35)	0.123	−0.64 (−1.05, −0.22)	0.036
*HOXA5*	Homeobox A5	−1.40 (−2.61, −0.18)	0.189	−1.93 (−2.50, −1.35)	0.0001
*HOXA7*	Homeobox A7	−1.54 (−2.78, −0.29)	0.174	−0.77 (−1.14, −0.40)	0.006
*HOXA9*	Homeobox A9	NA	NA	−3.31 (−4.66, −0.96)	0.002
*HOXB6*	Homeobox B6	0.74 (ND, ND)	0.685	0.92 (0.32, 1.52)	0.075
*ID2*	Inhibitor of DNA-binding 2	−0.34 (−1.31, 0.64)	0.699	0.59 (0.21, 0.96)	0.063
*JAM2*	Junctional adhesion molecule 2	−0.62 (−2.06, 0.82)	0.638	−0.91 (−1.32, −0.49)	0.004
*JAM3*	Junctional adhesion molecule 3	−0.55 (−1.58, 0.49)	0.566	−1.09 (−1.50, −0.68)	0.001
*KRT17*	Keratin 17	0.06 (−0.65, 0.77)	0.948	−1.63 (−2.56, −0.71)	0.013
*MME*	Membrane metallo-endopeptidase	−0.44 (−1.35, 0.46)	0.611	−4.42 (−5.58, −3.25)	<0.001
*PDGFA*	Platelet-derived growth factor receptor, alpha polypeptide	−0.98 (−1.77, −0.18)	0.174	−1.28 (−1.94, −0.63)	0.008
*SNAI2*	Snail homolog 2	−0.65 (−1.58, 0.29)	0.439	−1.79 (−2.59, −0.99)	0.004
*TGFBR3*	Transforming growth factor receptor, beta 3	−0.85 (−1.65, −0.05)	0.229	−2.02 (−3.09, −0.96)	0.010
*TJP3*	Tight junction protein 3	−0.47 (−0.99, 0.04)	0.294	1.13 (0.51, 1.76)	0.013
*WNT5B*	Wingless-type MMTV integration site family, member 5B	0.21 (−0.86, 1.27)	0.864	−2.71 (−3.75, −1.66)	0.001

HELU, hyperplastic enlarged lobular unit; TDLU, terminal duct lobular unit; ADH, atypical ductal hyperplasia; DCIS, ductal carcinoma *in situ*; HN, histologically normal; FDR, false discovery rate; CI, confidence interval; vs., versus; ND, not determined; NA, probe set not available on HG-U133A Human GeneChip microarray.

## Discussion

Overall, the pattern of gene expression in HELUs was consistent with the lobuloalveolar phenotype that characterizes this lesion. In fact, HELUs demonstrated the marked underexpression of genes typically related to the maintenance of a stem-like state, and the concomitant overexpression of genes specifically involved in forming the lobuloalveolar structure. Indeed, genes notably underexpressed in HELUs include *ABCG2* and *PROM1*, which code for 2 stemness-associated markers [15,16], and *NOTCH3* and *JAG2*, which encode Notch receptor 3 and Notch receptor ligand Jagged 2, respectively, 2 elements of the Notch signaling pathway that play a pivotal role in promoting a stem-like state in tumors with a basal phenotype [17,18]. Notably, concomitant underexpression of *MYC* corroborates the direct relationship between the JAG2-Notch receptor signaling pathway and c-Myc activity*.* The Notch signaling, in fact, promotes cell survival by regulating the expression of c-Myc transcription factor [19], which in turn, regulates the expression of *JAG2* [20] according to a feed-forward-loop transcriptional network. Other genes also underexpressed in HELUs include *SOX9* and *SOX10*, which encode two members of the sex-determining region Y-related (SOX) gene family of transcription factors. The proteins these genes encode are involved, as part of a highly coordinated transcriptional program, in maintaining the stem-like state and in activating the epithelial-to-mesenchymal transition [21,22]. Specifically expressed in myoepithelial cells and breast basal-like carcinomas [23], *SOX9* and *SOX10* have proven to be negatively associated with *FOXA1*, which encodes FoxA1 transcription factor, an essential regulator of mammary ductal morphogenesis [24]. Consistent with this evidence, we found that underexpression of *SOX9* and *SOX10* was associated with *FOXA1* overexpression in HELUs. In contrast with *SOX9* and *SOX10* underexpression, *SOX4*, another member of SOX gene family that plays a role in commitment to the differentiated luminal phenotype and to be progesterone-regulated [25], was constitutively expressed in HELUs.

In keeping with the physiologic role of progesterone in mammary gland branching morphogenesis [26], a key gene overexpressed in HELUs was *PGR*, which encodes progesterone receptor, the nuclear ligand-activated receptor required for the canonical genomic mechanism of action of progesterone. Notably, in addition to progesterone receptor, HELUs constitutively expressed *PGRMC1* and *PGRMC2*, which encode progesterone receptor membrane component 1 and 2, respectively. These proteins mediate the rapid transduction of the progesterone-induced signaling through a non-genomic mechanism of action [27]. Taken together, these findings support the progesterone dependence of HELUs and explain the peculiar hyperplastic aspect of this lesion.

Our results indicated that *HOXB2, HOXC10*, and *HOXC11* were overexpressed in HELUs*.* Interestingly, the proteins these genes encode are involved in hormone-dependent cell differentiation. *HOXB2* is a downstream target of retinoic acid, a well-known differentiation inducer required to maintain the homeostasis of mammary gland morphogenesis [28], and once activated, *HOXB2* encodes a transcription factor that negatively regulates growth in breast cancer cells [29]. Similarly, *HOXC10* and *HOXC11* are transcriptionally regulated sex hormone steroids [30,31], thus justifying their overexpression in HELUs. By contrast, we surprisingly observed underexpression of *ELF5*, which encodes ELF5, an epithelial-specific transcription factor that plays a crucial role as lineage gatekeeper during normal mammary development by driving the formation of lobuloalveoli from luminal progenitors [32,33]. Notably, we conducted another study of the same genes investigated here, but in normal bipotent progenitors, luminal precursors, and differentiated myoepithelial and luminal differentiated cells [34]. Consistent with the published role of ELF5, we found a positive association between *ELF5* expression and luminal commitment and differentiation as well as a negative association between *ELF5* expression and myoepithelial cells [34]. Hence, the unexpected underexpression of *ELF5* that we observed in HELUs suggests that silencing this gene, possibly via promoter methylation, may be an early event in pathologic transformation. Present results, however, seem to indicate a mechanism of silencing distinct from promoter methylation, as suggested by the underexpression of pivotal genes that function as transcriptional repressors in DNA methylation (*EZH1*) [35], histone modification (*MLL4*) [36], or chromatin remodeling *(SMARCA4*) [37]. A recent study provides an intriguing alternative explanation, demonstrating that progesterone drives mammary differentiation through the receptor activator of necrosis factor kappa B (RANK) ligand-mediated induction of ELF5 in luminal progenitor cells [38]. In fact, in the absence of progesterone, the luminal compartment is composed of mature *sensor* cells, which express PGR but not ELF5, and mature *secretory* cells, which express ELF5. After binding its receptor, progesterone induces sensor cells to secrete RANK ligand, the pivotal paracrine mediator of progesterone function [39]. RANK ligand, through ELF expression, then induces epithelial stem cell proliferation and forces asymmetric expansion and differentiation toward the secretory cell lineage of luminal progenitor cells. Present results fit this model well and suggest that HELUs are mainly composed of sensor cells characterized by the concomitant overexpression of *PGR* and underexpression of *ELF5*.

Considering the well-differentiated phenotype of HELUs, another apparent paradox was the underexpression of *ITGA6* and *ITGB4*, which respectively encode α6 and β4 integrin, the two main components of laminin receptor [40]. However, findings from several immunohistochemical studies provide a convincing explanation for the down-regulation of these adhesion molecules. In the normal adult breast, α6 and β4 integrins are primarily expressed on the basal surface of myoepithelial cells, where they anchor the underlying basement membrane, and are weakly expressed at the basolateral surface of luminal cells [41]. More detailed studies have shown that cells inside the lobule, which is not surrounded by a basement membrane, do not express α6 and β4 integrins [42], corroborating the underexpression of *ITGA6* and *ITGB4* that we observed in HELUs.

Finally, our results are also consistent with a differentiated luminal phenotype in HELUs. We observed underexpression of genes encoding growth factors (*EGF*, *MFGE8*, *TGFA*, and *TGFB2*) and transcription factors (*FOXC1*) that are primarily expressed in normal myoepithelium. These genes are also overexpressed in basal-like breast cancers (especially the triple-negative subtype), where they are involved in cell survival, neoangiogenesis, epithelial-to-mesenchymal transition, and migration [43-47].

In the gene expression profile for ADHs and DCISs relative to histologically normal tissue, we found a paradoxical association between terminal luminal differentiation (indicated by underexpression of basal marker genes and overexpression of *ESR1*) and cell proliferation (indicated by overexpression of *CCNB1*, *CCNB2*, and *MKI67*). The finding is particularly significant in understanding the neoplastic transformation process because it contradicts the notion that ER expression and cell proliferation are two dissociated, if not antithetic, processes in normal luminal cells [48]. Per this dissociation principle, estrogen induces never-dividing, ER-positive cells to produce growth factors that stimulate proliferation of adjacent, ER-negative cells in a paracrine manner [49]. Therefore, concomitant overexpression of *ESR1*, *CCNB1*, *CCNB2*, and *MIK67* supports the hypothesis that disrupting the mechanism governing the dissociation between ER expression and cell proliferation, and consequently establishing autocrine ER signaling [50], could be a crucial event in the transition from ADHs to DCISs, and provides a molecular explanation of why DCISs have a higher risk of progressing toward invasive cancer. Constitutive expression of ER allows ADH/DCIS-forming cells to exploit, in an autocrine manner, the proliferative stimulus induced by estrogens that bypass the constraint of the ER-proliferation dissociation, and promotes a dividing, ER-dependent, luminal phenotype. Notably, ADH/DCIS-forming cells were characterized by overexpression of *FOXA1* and *GATA3*, which encode two transcription factors (FoxA1 and Gata-3, respectively) that play a pivotal role in estrogen-regulated luminal differentiation and ductal elongation [24,51]. In particular, Gata-3, corecruited with FoxA1 to ER *cis*-regulatory elements, is essential for the ER-mediated transcription of target genes as part of a positive feedback loop in which ER expression is required for *GATA3* gene transcription [52]. Although FoxA1 and Gata-3 are recognized pioneer transcription factors, their binding capability depends on chromatin modifications. Therefore, the observation that *FOXA1* and *GATA3* overexpression was associated with *ACTL6A* and *EZH2* overexpression was not surprising, as *ACTL6A* and *EZH2* respectively encode a regulatory component of many ATP-dependent chromatin-remodeling complexes and the inducible catalytic subunit of polycomb repressive complex 2. However, studies in transgenic mice demonstrated that aberrant overexpression of *EZH2* is associated with disruption of ductal morphogenesis and promotion of hyperplastic epithelium that is predominantly composed of differentiated luminal cells expressing ER and high levels of Gata-3 [53].

We observed a dramatic decrease (up to 90% in DCISs respect to normal tissue) in *MME* expression in ADHs/DCISs. *MME* encodes CD10, a membrane metallopeptidase prevalently expressed in myoepithelium [54]. In normal tissue, myoepithelial cells control mammary gland homeostasis by forming a physical barrier between epithelial cells and the surrounding stroma and secreting paracrine mediators that inhibit cell migration [55]. Because the transition from *in situ* to invasive carcinoma is associated with the loss of myoepithelial layer, it is conceivable that the progressive underexpression of *MME* we observed in ADHs and DCISs represents a very early event in the process of malignant transformation.

When we compared genes that characterize HELUs with genes that characterize ADHs/DCISs, we found that HELUs and ADHs/DCISs shared only 5 genes despite their common luminal origin: *EGF*, *ELF5*, *FOXC1*, *JAG2*, and *SOX10*. Each of these genes was underexpressed relative to corresponding normal tissue. The underexpression of all but *ELF5* was expected. In fact, *EGF* underexpression is consistent with experimental evidence that epidermal growth factor supports the development of bipotent cells into both luminal and myoepithelial lineages but decreases during luminal lineage differentiation [56]. Similarly, *FOXC1* is specifically expressed in the myoepithelium and overexpressed in ER-negative breast cancers such as the basal-like type, in which it promotes the expression of genes involved in the epithelial-to-mesenchymal transition and enhances the propensity to metastasize [57]. Interestingly, recent studies have demonstrated that basal-like breast cancer pathogenesis can be inhibited by the repressive activity of Gata-3 on *FOXC1* expression [58] and by the silencing activity of EZH2 [59]. Our results and the aforementioned results show that in both types of precursors (HELUs and ADHs/DCISs), the underexpression of *FOXC1* is associated with the overexpression of *GATA3* and *EZH2*, suggesting that *GATA3* and *EZH2* protect against the transition to an invasive mesenchymal phenotype triggered by *FOXC1* overexpression. The underexpression of *JAG2* and *SOX10* was also unsurprising, because these genes*,* as previously discussed, are involved in promoting and maintaining the stem-like state. Notably, both genes are specifically expressed in myoepithelial cells and in breast cancers with a basal phenotype [17,21]. In regard to *ELF5*, underexpression interestingly appears to be progesterone-dependent in HELUs but caused by gene promoter methylation in ADHs/DCISs (as suggested by the concomitant overexpression of *EZH2*).

## Conclusions

Although our study has limitations common to the majority of studies involving only gene expression profiling, i.e., small number of cases and lack of validation of the observed mRNA modulations at the protein level, the results we report here provide interesting information. Despite their common luminal origin, HELUs and ADHs/DCISs are distinct entities, characterized by different gene profiles with very little overlap. In particular, terminal lobular differentiation of HELUs is characterized by overexpression of *PGR* and constitutive expression of *PGRMC1* and *PGRMC2* (suggestive of a progesterone-dependence), whereas the terminal ductal differentiation of ADHs is characterized by overexpression of *ESR1* and *GATA3* (suggestive of an estrogen-dependence) and by the unexpected concomitant overexpression of *CCNB1*, *CCNB2*, and *MKI67.* The latter finding is particularly interesting because it supports early disruption of the mechanism governing the dissociation between ER expression and cell proliferation, and the consequent establishment of autocrine ER signaling.

Although clinical evidence indicates that only a small fraction of HELUs and ADHs evolve to invasive cancer, the present findings suggest that exposure to synthetic progestins, frequently administered as hormone-replacement therapy, and estrogens, when abnormally produced by adipose cells and persistently present in the stroma surrounding the mammary gland, must be regarded as potential causes for the development of these hyperplastic lesions.
